# Earlier relapse detection after allogeneic haematopoietic stem cell transplantation by chimerism assays: Digital PCR versus quantitative real-time PCR of insertion/deletion polymorphisms

**DOI:** 10.1371/journal.pone.0212708

**Published:** 2019-02-22

**Authors:** Jennifer Valero-Garcia, María del Carmen González-Espinosa, Manuel Barrios, Greta Carmona-Antoñanzas, Javier García-Planells, Carlos Ruiz-Lafora, Ainhoa Fuentes-Gálvez, Antonio Jiménez-Velasco

**Affiliations:** 1 Instituto de Medicina Genómica (imegen), Paterna, Valencia, Spain; 2 Haematology Department, Hospital Regional Universitario de Málaga, Instituto de Investigación Biomédica de Málaga (IBIMA), Málaga, Spain; European Institute of Oncology, ITALY

## Abstract

**Background:**

The analysis of molecular haematopoietic chimerisms (HC) has become a well-established method to monitor the transplant evolution and to assess the risk of relapse after allogeneic stem cells transplantation (allo-STC). Different techniques and molecular markers are being used for chimerism surveillance after transplantation, including quantitative real-time PCR (qPCR) and the recently developed digital PCR (dPCR). This study aims to compare the sensitivity and accuracy of both methods to quantify HC and predict early relapse.

**Methodology:**

HC was evaluated using custom PCR systems for the specific detection of the Y-chromosome, null alleles and insertion-deletion polymorphisms. A total of 281 samples from 28 adult patients who underwent an allo-SCT were studied. Increasing mixed chimerism was detected prior to relapse in 100% of patients (18 relapses).

**Results:**

Compared with conventional qPCR amplification, dPCR predicted relapse with a median anticipation period of 63 days versus 45.5 days by qPCR. Overall, 56% of the relapses were predicted earlier with dPCR whereas 38% of the relapses where detected simultaneously using both techniques and only in 1 case, relapse was predicted earlier with qPCR.

**Conclusions:**

In conclusion, chimerism determination by dPCR constitutes a suitable technique for the follow-up of patients with haematological pathologies after allo-STC, showing greater sensitivity to predict an early relapse.

## Introduction

Allogeneic Stem Cell Transplantation (allo-SCT) is considered to be the strongest immunotherapy for a variety of malignant and non-malignant hematologic disorders. Despite its strong curative potential and improved safety, allo-SCT remains a high-risk procedure [1]. Haematopoietic chimerism (HC) analysis, distinguishing recipient from donor cell subsets after allo-SCT, has become an important and well-established method to monitor the graft health, predict early detection of disease relapse and provide useful information on the graft-versus-host disease (GvHD) or graft-versus-tumor (GvT) effect, facilitating therapeutic intervention [2].

Traditionally, the study of HC has been performed using cytogenetic strategies such as fluorescent *in situ* hybridisation (FISH) of blood and bone marrow cells [3], and molecular analysis of VNTR (Variable Number of Tandem Repeats) or STR (Short Tandem Repeat) polymorphisms by conventional polymerase chain reaction (PCR) and fragment analysis [4–6], currently considered the gold standard on chimerism surveillance after transplantation [7]. In studies of leukaemia patients undergoing an allo-SCT, the sensitivity of the chimerism analysis using STR-PCR methods was incapable to detect values below 1% receptor of blood cells in total peripheral blood (PB), whereas slightly improved sensitivities were observed when cell separation of CD34^+^ or specific leukocyte subsets were isolated from PB or bone marrow samples [4,8,9]. However, neither of these strategies provides sufficient sensitivity and quantitative accuracy to be considered the optimal methodology to detect increasing mixed chimerism (iMC) for efficient prediction of early morphological relapse [10].

Over a decade ago, Alizadeh et al. [11] proved that using quantitative real-time PCR (qPCR) of single nucleotide polymorphisms (SNPs) to monitor HC resulted in considerably improved sensitivity, detecting up to 0.1% host DNA in whole blood samples [11]. Later, a novel qPCR approach based on more robust insertion-deletion (INDEL) polymorphisms was published. With this strategy, the sensitivity was significantly improved, and acute leukaemia relapse was anticipated earlier in 88% of cases versus 44% when fragment analysis were used [12]. Chimerism analysis by qPCR has proven to be superior in sensitivity; however, the technical error inherent to the method can result in a substantial deviation from the true value (up to 25% variation), which might be therapeutically significant in cases of persistent mixed chimerism [10]. Whereas the use of replicates can slightly minimise the technical error, the inclusion of previous follow-up samples is recommended in each determination, and the construction of a standard curve, generally using the pre-transplantation samples, is required to evaluate the monitor the patient status [13,14].

A recently developed technology, known as digital PCR (dPCR), is currently on the spotlight of a broad range of medical specialties as it offers the potential to perform absolute quantification accurately, adding improved sensitivity and precision to the traditional molecular methods [15–17]. Digital PCR enables absolute quantification by partitioning of the sample in thousands of functional sub-replicates, thus eradicating the need to use replicates and calibration curves. Moreover, dPCR has been shown greater accuracy and robustness in the presence of PCR inhibitors, saving inherent technical limitations of qPCR [18,19].

Recent studies have suggested that dPCR allowed earlier mixed chimerism (MC) detection compared to STR-PCR analysis [10,20,21]; however, the clinical potential of dPCR in HC surveillance is yet scarce and further research is needed to establish the clinical relevance of digital PCR versus conventional molecular technologies. We hypothesise that dPCR could replace previous methods of HC monitoring and predict relapse earlier due to its greater accuracy. In addition, its greater precision makes this technique suitable to monitor the patient and transplant health from very low values of receptor blood cells, close to complete chimera, to persistent mixed chimerism. Thus, the aim of this work was to perform a comparative study between qPCR and dPCR in the hematopoietic chimerism analylsis of 28 leukaemia patients undergoing allo-SCT.

## Materials and methods

### Patient characteristics

The present study was approved by the Hospital Universitario de Málaga research ethics committee. Clinical data was collected from each patient after written informed consent was obtained and reviewed by the ethics committee. Signed informed consents were recorded a part of the patient’s clinical records.

Twenty-eight patients with haematologic malignant diseases treated with allo-SCT were included in this study. Underlying diseases were Myelodysplastic Syndrome (MDS-RAEB-II; n = 3), Acute Myeloid Leukaemia (AML; n = 15), Acute Lymphoblastic Leukaemia (ALL, n = 8) and Chronic Lymphocytic leukaemia (CLL; n = 2) (Table 1).

**Table 1:** Information of the allo-SCT patients included in the study.

Patient	Sex	Leukaemia type	Marker	Relapse
Patient 1	M	MDS	SRY	YES
Patient 2	M	AML	Q116-6I	NO
Patient 3	M	AML	Q116-10I	YES
Patient 4	F	MDS	Q116-11I	YES
Patient 5	M	ALL	Q116-12I	YES
Patient 6	M	AML	Q116-10I	YES
Patient 7	M	AML	Q116-4I	YES
Patient 8	F	ALL	Q116-31I	NO
Patient 9	F	AML	Q116-7I	NO
Patient 10	F	ALL	Q116-6I	YES
Patient 11	F	ALL	Q116-3I	YES
Patient 12	F	AML	Q116-8I	NO
Patient 13	M	AML	Q116-32I	NO
Patient 14	M	AML	Q116-11I	YES
Patient 15	F	AML	Q116-6I	YES
Patient 16	M	AML	Q116-31I	YES
Patient 17	M	AML	Q116-12I	NO
Patient 18	F	AML	Q116-6I	YES
Patient 19	F	ALL	Q116-9I	YES
Patient 20	M	ALL	Q116-8I	NO
Patient 21	M	ALL	SRY	YES
Patient 22	F	ALL	Q116-11I	YES
Patient 23	M	MDS	Q116-5I	YES
Patient 24	M	AML	Q116-11I	NO
Patient 25	M	AML	SRY	YES
Patient 26	M	CLL	SRY	NO
Patient 27	F	AML	Q116-4I	YES
Patient 28	M	CLL	Q116-6I	NO

MDS, Myelodysplastic Syndrome; AML, Acute Myeloid Leukaemia; ALL, Acute Lymphoblastic Leukaemia; CLL, Chronic Lymphocytic leukaemia; ND, not defined

### Patient samples

A total of 281 post-allo-SCT peripheral blood samples were included in the clinical study, of which 56 samples corresponded to the donor/recipient pairs and 225 samples to post-allo-SCT follow-up samples (3 of which resulted in no valuable data due to low quality sample). Patients were routinely investigated for chimerism every 10–15 days during the first three months post-transplant, and monthly until the completion of the first year. Beyond the first year patients were monitored for chimerism at 3 months intervals. Additional samples for chimerism testing were performed based on the presence of high-risk factors and on clinical grounds.

Increasing mixed chimerism (iMC) is the main indicator of increased risk of graft loss and relapse, generally defined as the significant increase in the proportion of host cells (or decrease in percentage of donor cells) in PB between two consecutive assessments having stablished the threshold at 0.1% host blood cells. That is, iMC occurs when ≥ 0.1% recipient cells are detected following a previous lower value, or an increase of at least 0.1% above the 0.1% threshold is detected [22]. After the allogeneic stem cell transplantation, the health status of each patient was monitored by optical microscopy. For this, bone marrow samples were routinely collected on day +30 post-allo-SCT. Additional bone marrow samples were collected when iMC was detected under the suspicion of high-risk of relapse or graft rejection. Complete remission was confirmed when fewer than 5% blast cells were found in regenerated bone marrow, whereas relapse was diagnosed when more than 5% blast cells were observed in bone marrow, or extramedullary leukemic cells appeared in patients with acute leukaemia or myelodysplastic syndromes (myelodysplastic syndrome-refractory anemia with excess blasts type-II (MDS-RAEB-II). In patients with chronic lymphatic leukaemia (CLL) the relapse was diagnosed when B-cell clonality was detected by FACS analysis.

### Genomic DNA extraction

Post-transplantation PB samples were processed immediately after collection and genomic DNA extracted from 400 μl of PB collected in EDTA tubes for the follow-up analysis using the Maxwell 16 Blood DNA purification Kit (Promega, Madison, USA). DNA samples were quantified with NanoDrop spectrophotometer (ThermoFisher Scientific) and stored at -80°C until all the post-allo-SCT samples had been processed and PCR analyses were performed. In addition, donor blood samples and receptor pre-transplantation samples had been previously collected and processed following the aforementioned extraction procedure in order to perform the screening assays required to select informative markers.

### Haematopoietic chimerism assays: Screening assays

Prior to the follow-up analysis, one informative marker was selected for each patient among the 18 available markers, including di-allelic INDELs, Y-chromosome-specific maker in case of sex-mismatched allo-SCT from the SRY gene, and null alleles. For HC studies after allo-SCT, genetic markers are considered informative when they are detected in the recipient (pre-transplant) sample and absent from the donor sample. The genotyping of host and donor samples was performed by real-time PCR following the manufacturer’s protocol (imegen-Quimera Screening, imegen).

### Haematopoietic chimerism assays: Follow-up assays

The monitoring HC studies were performed using genomic DNA samples and imegen-Quimera commercial kits, including custom qPCR and dPCR systems for the detection of Y-chromosome specific markers and INDEL di-allelic polymorphisms markers and/or null alleles. The oligonucleotides and hydrolysis probes used in the chimerism studies were provided by Instituto de Medicina Genómica (imegen) and used according to the manufacturer’s recommended protocol. The sensitivity of these systems had been previously estimated during the validation of imegen-Quimera. The limit of detection (LOD), understood as the lowest amount of host blood cells reliably detected by the system was 0.01% for both qPCR and dPCR. The limit of quantification (LOQ), defined as the lowest amount of host blood cells that can be reliably quantified (within a variation coefficient < 25%), varied significantly between both techniques, accounting for 0.1% by qPCR and 0.05% by dPCR (Validation reports provided upon request). The polymorphic markers included in the screening and quantification of HC analysis were bioinformatically retrieved according to the following criteria: placed outside repetitive regions, di-allelic polymorphisms and described frequency for the most common allele in the population ~ 0.7. The aim of this bioinformatics filter was to achieve the maximum informativity in the population using the fewest possible markers. The cumulative informativity of the 18 analysed markers was 99.1%.

### Real-Time PCR Monitoring (qPCR)

The relative quantification of HC was performed using a reference gene encoding for the β-globin protein and one informative polymorphism. Target assays and β-globin were analysed in the same run into separate reactions using target-specific oligonucleotides and FAM–labeled hydrolysis probes. Relative quantification of informative markers was performed according to a previously described protocol [23]. All samples were analysed in duplicate and PCR conditions were the same for all the polymorphisms. Thermal cycler program conditions consisted of an initial denaturation step at 95°C for 10 min, followed by 45 amplification cycles consisting of denaturation at 95°C for 10 sec and annealing and extension at 58°C for 25 s, and one final step at 40°C for 15 s.

### Digital PCR Monitoring (dPCR)

Digital PCR analysis was carried out using the QuantStudio 3D Digital PCR System (ThermoFisher Scientific). In this case, both the reference gene, β-globin and the informative marker were multiplexed in the same PCR reaction, using hydrolysis probes labeled with VIC for the β -globin, and FAM for the polymorphic marker. PCR conditions were the same for all analysed markers. A total of 120 ng of genomic DNA (6 μL at 20 ng/μL) were used in combination with 0.9 μM of specific oligonucleotides (Forward and Reverse for each of the two targets, β -globin and polymorphic marker), 0.25 μM each hydrolysis probe (FAM and VIC) and 7.5 μL of Applied Biosystems QuantStudio 3D Digital PCR Master Mix v2 (Catalog Number: A26358;ThermoFisher Scientific). From this mix, a total of 14.5 μL was loaded into the chip by the QuantStudio 3D Digital PCR Chip Loader (ThermoFisher Scientific) and subsequently sealed and loaded on the Gene Amp 9700 PCR System. The PCR program conditions included an initial step at 45°C for 60 s, followed by and initial denaturation step at 98°C for 150 s, and 40 amplification cycles consisting of: 98°C for 60 s, 56°C for 10 s and 60°C for 30 s. Chips were stored in the dark at room temperature for 45 min prior to reading using the QuantStudio 3D Digital PCR instrument (ThermoFisher Scientific).

Recipient chimerism was calculated using copies/μL obtained from the data analysis software QuantStudio 3D AnalysisSuite Cloud software (v3.1), as it is shown below.

Recipient chimerism (%) = [(Target copies/μL) / (Reference copies/μL)] x 100 Number of reactions containing each target was calculated by Poisson-Plus Model distribution estimates, using the number of non-target containing reactions relative to the total number of reactions [22].

### Statistical analyses

To confirm the validity of the study and compare between both quantification techniques, the correlation between both quantifying methods was calculated. For that, Pearson correlation coefficient (ρ_X,Y_) was computed to measure the grade of relationship between the two quantitative variables (qPCR and dPCR). In addition, the square of the Pearson correlation coefficient (R^2^) was computed to determine the replicability of the model, and the quality and the variability of the results that can be explained by the model. Finally, a Bland-Altmann plot was used to visualise the grade of concordance between the results obtained by both, qPCR and dPCR methods.

## Results

### Statistical results

**Fig 1:**
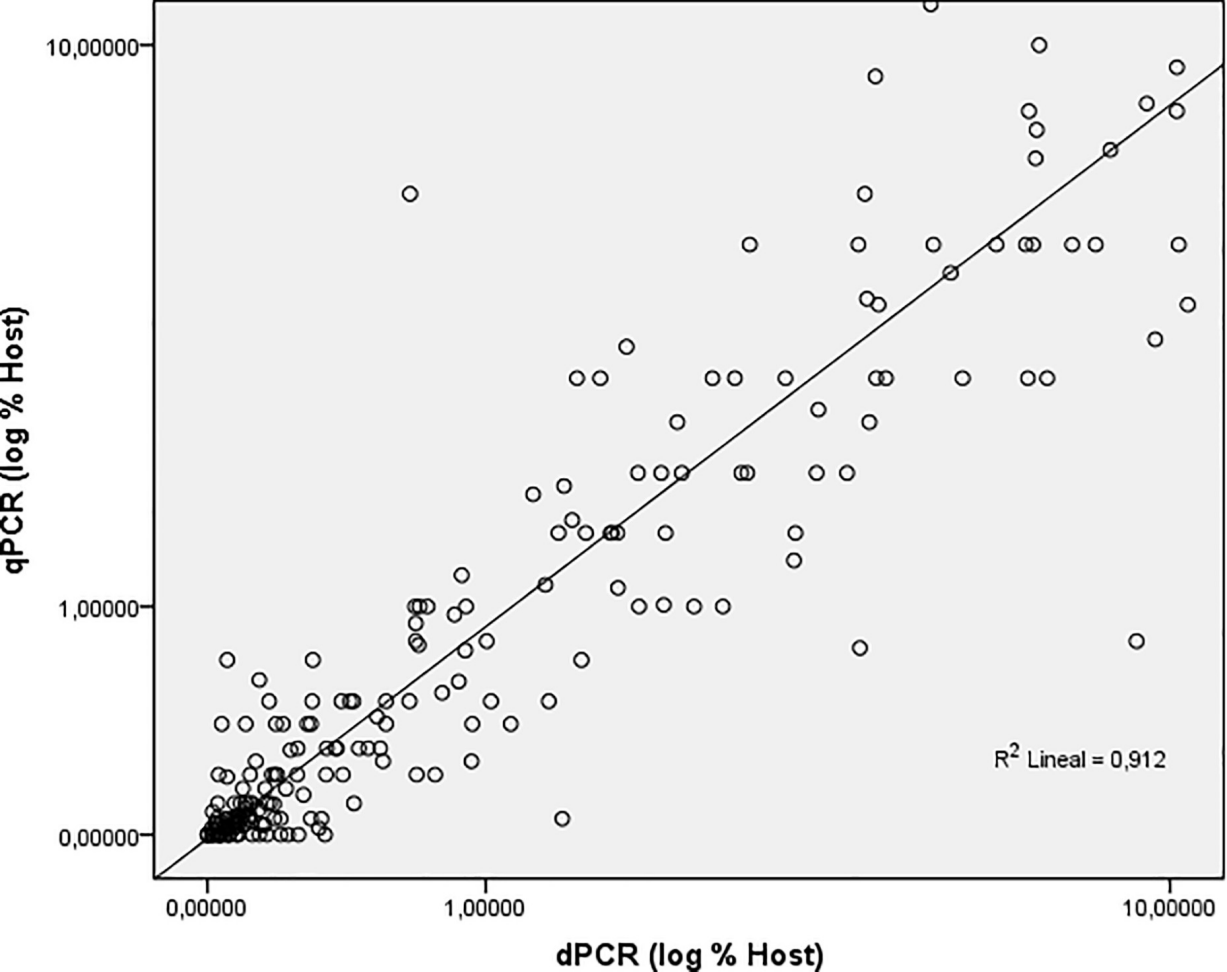
Correlation of hematopoietic chimerism data as obtained on clinical samples by dPCR versus qPCR. Chimerism was assessed for post-allo-SCT samples and obtained data were analysed for linear regression of logarithmic percent host chimerism. A very high degree of linear correlation (R^2^ > 0.91) was observed.

**Fig 2:**
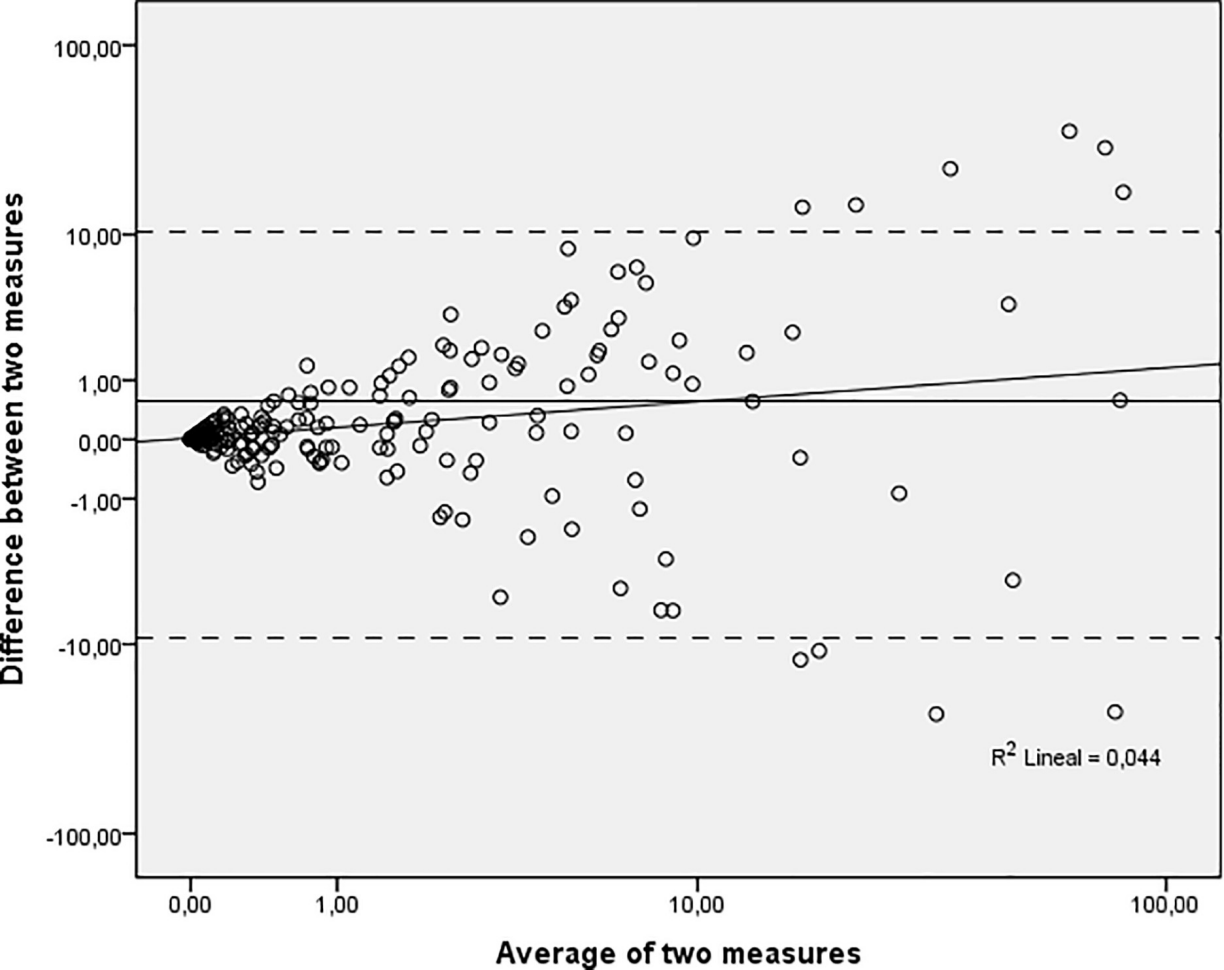
Bland-Altmann analysis of dPCR data as compared to qPCR. The difference between qPCR and dPCR quantification is plotted against their mean.

Pearson correlation coefficient (ρ_X,Y_) was calculated to confirm the validity of the study and comparability between both techniques, indicating that both quantitative variables were positively correlated (R = 0.959). Similarly, the square of the Pearson correlation indicated a suitable repeatability of the results, accounting for R^2^ = 0.91, with only a few diverging values (Fig 1). In addition, a Bland-Altmann graphic was plotted to visualise the concordance rate between qPCR and dPCR methods. The difference mean value was 0.56 (standard error: 0.33) with a confidence level of 95% and a confidence interval ranging between -9.22% and 10.34%, thus suggesting a close data correlation between the two techniques (Fig 2). Overall, these results indicate the validity of the study, showing high correlation between both methods and ensuring the relationship between both techniques for the study of mixed chimerism.

### Chimerism determination

Screening of recipient and donor samples showed informative markers for all the 28 allo-SCT patients (Table 1). In order to test our initial hypothesis, all the peripheral blood samples corresponding to the 28 leukaemia patients were simultaneously analysed by qPCR and dPCR methods, using the same polymorphic marker throughout the whole follow-up study.

**Fig 3:**
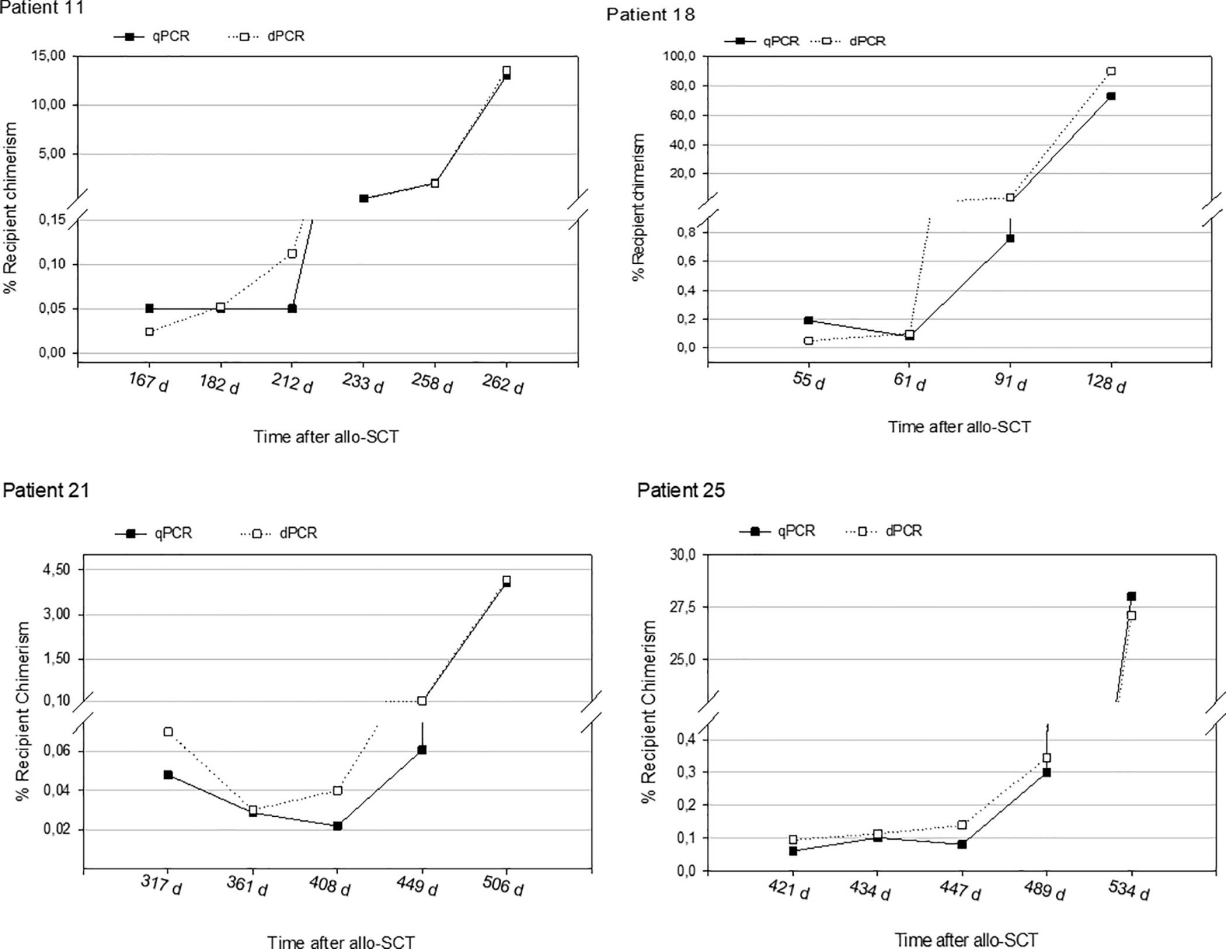
Comparison of chimerism kinetics in four relapsed patients of allo-SCT assessed by dPCR and qPCR. Follow-up analysis by qPCR and dPCR was carried out using the same host-specific marker. Data plotted represent the most informative time window in the follow-up study of each transplantation patient. iMC was detected earlier by dPCR in patients 11, 18, 21 and 25).

In our study, the analysis of chimerism was performed in all transplanted patients who relapsed, 18 patients out of the 28 allo-SCT cases included in the study. The chimerism pattern in the relapsed patients was clearly different from the pattern observed in the non-relapsed patients where an ascending trend was observed (Fig 3).

### Early-relapse prediction by dPCR and qPCR

In our series, the HC analysis indicated that iMC could be identified in all the relapsed patients (18 patients) prior to the haematologic relapse, characterised by blasts levels greater than 5% (optical microscopy or flow cytometry studies performed in bone marrow). Overall, iMC was detected earlier by dPCR in 55.6% of the total allo-SCT patients, whereas iMC was detected simultaneously by qPCR and dPCR in 38.9%, and only in one patient, iMC was identified earlier by qPCR (5.5%).

Overall, the median of time between the dPCR-detected iMC and the relapse was 63 days (range: Day 14 –Day 301) versus 45.5 days (range: Day 2 –Day 100) by qPCR. This indicates that the results obtained by dPCR elucidated a gradual increment of host blood cells, iMC, earlier than when qPCR was used. Thus, concluding that more than 50% of patients, who have relapsed, would have been previously detected if the HC analysis had been performed using dPCR.

## Discussion

Despite the continuous improvements in allo-SCT, relapse remains one of the main causes of failure of the procedure, especially in patients with acute leukaemia. HC analysis constitutes a standard procedure in the follow-up of patients undergoing allo-SCT capable to predict disease recurrence, graft-versus-host disease and graft failure. In a significant proportion of patients there is no minimal residual disease marker that allows us to monitor the risk of relapse, whereas the analysis of chimerism is possible in almost every patient. STR-PCR analysis is the gold standard for quantitative chimerism analysis recommended by the EuroChimerism consortium in the prediction of relapses [12,23]; however, its limited sensitivity, allelic imbalance and high coefficient of variation at low percentage of mixed chimerism has opened the ground to investigate novel technologies [12,20,24].

The advantages of qPCR-based methods over the conventional STR-PCR analysis have been demonstrated by multiple studies of HC chimerism surveillance [12,24]. Comparatively, qPCR is highly sensitive, capable to detect as little as 0.01% recipient cells (1 recipient cell among 10,000 of the donor), but it is highly influenced by the efficiency of every amplification reaction and calibration curves and replicate assays are required due to the limited technical precision. In addition, quantitative results should be normalised with respect to an endogenous gene amplified in separate PCR reactions. Digital PCR, on the other hand, offers a patient-specific approach that enables the multiplexed absolute quantification of target and endogenous genes in the same assay, standard curves are not needed and it is less sensitive to the action of PCR inhibitors given that it detects the target at the end point of amplification [25]. This simplifies the procedure by providing equivalent sensitivity values, but greater accuracy and lower variability in the quantification of HC at any level of detection.

Clinically, it is considered that a patient has a greater risk of relapse when a gradual increase in recipient cells is detected by molecular biology techniques, which does not disappear even when exposing the patient to different treatments like immunosuppression tapering or donor leukocyte infusion. This increase, known as iMC can occur weeks, months and even years after the allo-SCT took place, and it is therefore considered a symptom of potential relapse [26]. In this context, the accuracy of the dPCR technology has led other research teams to study the potential of dPCR in the surveillance of HC. Waterhouse et al. [20] discovered that the mean time from mixed chimerism detection to relapse was 155 days versus 65 days from dPCR and STR-PCR, respectively, and concluded that dPCR was a sensitive and accurate method for the quantification of hematopoietic chimerism that allowing earlier MC detection compared to STR-PCR. Similar studies highlighted the benefits of digital PCR over STR-PCR in the follow-up of allo-SCT patients [10,21], including a study in which the greater sensitivity resulted in MC detection in samples where complete donor chimerism was detected by STR-PCR [27].

In the present study, we studied 28 patients of allo-SCT using dPCR and qPCR to determine the feasibility to standardise the use of dPCR as a model method for mixed chimerism monitoring. In line with previous studies, our findings suggest that dPCR might be a more suitable method to use for the detection of haematopoietic chimerisms in the follow-up period [10,20,21]. Overall, our results indicated that both qPCR and dPCR could predict the relapse in peripheral blood before it was detected by optical microscopy; however, the highlight was that in the majority of the relapsed allo-SCT patients the first host cell increase (iMC), indicative of a greater risk of relapse, was detected on average 17.5 days earlier by digital PCR. In addition, the molecular results obtained by dPCR confirm the superior sensitivity of the technology as it enabled to detect informative marker increments from 10^−4^ to 10^−3^, expressed on a logarithmic scale. This is in accordance with a comparative chimerism study [12], which concluded that relapse prediction might be more informative if performed using peripheral blood samples given than the presence of residual recipient cells in bone marrow implies a status of complete chimerism cannot be achieved in bone marrow.

The analytical accuracy and precision of dPCR play a crucial role in the study of molecular chimerism enabling to reliably differentiate between a HC increase and an oscillation of the target values at a level of 10^−3^ and to distinguish the host cell increase weeks earlier that using qPCR. Clinicians, on the basis of the results of chimerism analysis, make major therapeutic decisions [1], and that there is long standing evidence indicating that early detection of MC, followed by the appropriate therapeutic action (i.e. dosing and withdrawal of immunosuppression post-HSCT, transfusion of donor lymphocytes or other cellular therapy, administration of immunomodulatory cytokines) can improve disease outcome after SCT [27]. In conclusion, our results indicated that dPCR performed on peripheral blood constitutes an adequate approach for the detection of mixed chimerism that enables faster prognosis of early relapse compared to qPCR, and might subsequently, effect favourably on the health and survival of allo-SCT patients.

## Supporting information

S1 Table Chimerism data for the 28 allo-SCT patients. Follow-up analysis by qPCR and dPCR was carried out using the same host-specific marker.
(XLSX)
