# Correction: Earlier relapse detection after allogeneic haematopoietic stem cell transplantation by chimerism assays: Digital PCR versus quantitative real-time PCR of insertion/deletion polymorphisms

**DOI:** 10.1371/journal.pone.0213966

**Published:** 2019-03-13

**Authors:** Jennifer Valero-Garcia, María del Carmen González-Espinosa, Manuel Barrios, Greta Carmona-Antoñanzas, Javier García-Planells, Carlos Ruiz-Lafora, Ainhoa Fuentes-Gálvez, Antonio Jiménez-Velasco

## Notice of republication

Incorrect versions of Table 1 and S1 Table were published in error. This article was republished on March 1, 2019 to correct for these errors. Please download this article again to view the correct version.
